# Home Training

**Published:** 1872-11

**Authors:** 


### HOME TRAINING.

The home education of our daughters, who are to be the mothers of the next generation, is a subject which commends itself to old and young; and any practical hints in regard to it (such as we give below) should be carefully considered, especially by every mother of a family of girls. Many young wives have been rendered miserable, and their newly found homes unhappy, because an over-indulgent mother had always attended to the domestic affairs of her own household herself, and brought her daughters up to be parlor ornaments, instead of dividing with them these labors and cares, and by this means qualifying them to perform these duties when they leave the maternal roof for one of their own. Not a reader of this Journal but may put a finger upon a score of cases where the young wife has endured years of misery for the lack of this very instruction and practice, which her mother should have given her at home. How this condition of things is to be remedied, is a question of the deepest interest to every woman in the country. The following plan, adopted by a sensible mother, commends itself to our judgment, as one well worthy of being followed by every mother in the land. Some days since, a friend called at our office, having read an article in a previous number, and in reference to it, related the following story: —

"When I was a young man," said he, "I used to visit a family in a neighboring State, consisting of a widowed mother and three or four daughters, whose ages ranged from sixteen to twenty-five. The family was in comfortable circumstances, and the mother still continued the business which her husband had long ago established. The daughters had the best education the place afforded, and as the town or city in which they lived was the seat of a collegiate institution, the society of the place was far superior to that of ordinary country villages, and the parlors of this family were a favorite resort for all those students whose tastes were best gratified in the society of agreeable and cultivated young ladies.

"It was the practice of this good, Christian mother, when one of her daughters graduated from school, to enter her as a 'freshman' in the school at home, where she was required to learn the art and mystery of housekeeping, how to provide, how to cook, how to serve up, how to preside at table, how to take care of the house from garret to cellar; and after she was thoroughly trained, she was required to take her turn with her mother and sisters, week in and week out, in taking entire charge of the house, even to presiding at every meal at table.

"O, Mary, Sarah, Elizabeth, Fannie, have you not often had occasion, when you have heard of the suffering of other young wives, with their early trials of housekeeping, to call your dear mother 'blessed'?

"At the time I was in the habit of visiting there, the youngest daughter, Fannie, was just entering upon this phase of her education, and the ease and quiet dignity with which these sisters performed their duties, and presided at table, was the subject of remark to all the visitors at the house.

"I began by saying, 'When I was a young man,' and that was a long time ago, as these white hairs, bleached by the frosts of nearly threescore and ten winters can testify; and the forty or fifty years which have intervened since the days I am writing about, have been attended with many vicissitudes. I have seen and known many people and many parts of the world, but the memories which cluster around the family circle I am telling you of, always bring pleasure with them, and those days and scenes seem to form a beautiful oasis in my early life.

"These young ladies subsequently married, and all raised up families of children; and I don't believe they were either of them ever obliged to write a letter to Mrs. Beecher to tell of her ignorance of housekeeping, and to ask her advice, for they had a good, sensible woman for a mother, who knew what was the best possible dowry she could bequeath to her daughters.

"Here is an example worthy to be held up to the readers of this JOURNAL. If the mothers who read it will adopt the practice of our worthy and sensible friend, they will save their poor daughters a vast deal of anxiety and misery, and make them, of course, far more eligible as wives, whether they happen to marry rich or poor. But I apprehend the same result will attend the preaching of the JOURNAL, which my own preaching in private has met with. I have related this story, perhaps, to five hundred different mothers of families, and have yet to meet with the first one who did not highly approve of the plan; and I have also to meet with the first one who has adopted it!"

